# Hyperoside ameliorates NAFLD in rats via remodeling gut microbiota and reprogramming serum metabolic networks

**DOI:** 10.3389/fnut.2025.1741026

**Published:** 2026-01-12

**Authors:** Mingchun Huang, Ya Wang, Yanyan Li, Weiwei Zeng

**Affiliations:** 1 Department of Pharmacy, Chongqing Hospital of Traditional Chinese Medicine, Chongqing, China; 2 Department of Pharmacy, The Sixth People’s Hospital of Dongguan, Dongguan, China; 3 Department of Cancer Center, The Second Affiliated Hospital of Chongqing Medical University, Chongqing, China

**Keywords:** gut microbiota, hyperoside, lipid metabolism, liver fibrosis, metabolomics, non-alcoholic fatty liver disease (NAFLD)

## Abstract

**Objective:**

This study explores hyperoside’s therapeutic efficacy in non-alcoholic fatty liver disease (NAFLD) rats and its gut–liver axis mechanisms through integrated gut microbiota and metabolomics analyses.

**Methods:**

The SD rats were divided into five groups (normal control, NAFLD model, low-dose hyperoside [0.6 mg/kg/day], high-dose hyperoside [1.5 mg/kg/day], and rosiglitazone positive control [5 mg/kg/day]) and treated for 12 weeks. Body weight, serum biochemistry (ALT, AST, TC, TG), liver histopathology (H&E, Sirius Red), hepatic mRNA expression (*Tlr4, Tnf-α,* and *α-SMA*), gut microbiota (16S rRNA sequencing), and serum metabolites (untargeted metabolomics) were assessed.

**Results:**

Hyperoside dose-dependently reduced high-fat, high-sugar diet-induced body weight gain, liver index, and hepatic steatosis/fibrosis, lowered serum liver enzymes and lipid levels, and downregulated pro-inflammatory/fibrotic genes. It remodeled gut microbiota by enriching Lactobacillus and suppressing pathobionts (e.g., *Streptococcus*, *Escherichia-Shigella*), reversed metabolic disturbances (e.g., 3-hydroxybutyric acid, diacylglycerols), and targeted glycine/serine/threonine and alpha-linolenic acid metabolism. Beneficial bacteria were negatively correlated with pro-inflammatory metabolites like lysophosphatidylcholine.

**Conclusion:**

Hyperoside ameliorates NAFLD, which is associated with gut microbiota remodeling and modulation of host metabolic networks, supporting its potential as a multi-target therapeutic agent for NAFLD.

## 1 Introduction

Non-alcoholic fatty liver disease (NAFLD) is now a primary contributor to cirrhosis, hepatocellular carcinoma, and end-stage liver disease (1, 2). The disease ranges histologically from simple steatosis to non-alcoholic steatohepatitis (NASH), potentially progressing to liver fibrosis, cirrhosis, and hepatocellular carcinoma (3). Although the pathogenesis of NAFLD involves multiple interrelated mechanisms—including insulin resistance (4), dysregulation of lipid metabolism (5), mitochondrial dysfunction (6), and chronic low-grade inflammation (7)—no specific pharmacological therapy has been approved for clinical use. Current management strategies primarily rely on lifestyle modifications, which often yield limited efficacy and poor long-term adherence (8). Thus, investigating natural bioactive compounds capable of multi-target regulation offers a promising strategy for NAFLD prevention and therapy.

Recently, gut microbiota–host interactions have received growing attention in NAFLD pathogenesis and progression (9, 10). A high-fat and high-sugar diet disrupts gut flora and barrier function, promoting systemic translocation of toxins like lipopolysaccharide (LPS). These microbial components activate hepatic Kupffer and stellate cells, initiating inflammatory responses that are correlated with fibrogenesis (11–13). Furthermore, metabolites produced by the gut microbiota—such as short-chain fatty acids (SCFAs), bile acids, and trimethylamine N-oxide (TMAO)—are crucial in modulating host energy homeostasis and metabolic equilibrium (14, 15). These results indicate that modulating the gut microbiome could serve as a promising therapeutic approach for NAFLD.

Given this background, traditional Chinese medicine (TCM) compounds, known for their holistic “multi-component, multi-target, multi-pathway” approach, have shown distinct benefits in treating metabolic diseases. Hugan Qingzhi Formula, a clinically used TCM prescription for liver disorders, consists of several herbal components including *Crataegus pinnatifida*, *Nelumbo nucifera* leaf, *Salvia miltiorrhiza*, and *Artemisia capillaris*, and is traditionally employed to promote liver function, strengthen the spleen, activate blood circulation, resolve stasis, clear heat, and eliminate dampness (16) Previous pharmacological studies have shown that this formula exerts significant protective effects against experimental liver injury. Hyperoside (Hyp), a major flavonoid glycoside abundant in *Crataegus* and *Nelumbo* species, has been reported to possess potent anti-inflammatory (17), antioxidant (18), and anti-fibrotic activities (19), with demonstrated hepatoprotective effects in models of alcoholic and chemically induced liver injury (17, 20). Nevertheless, the possible involvement of Hyp in NAFLD has not yet been investigated.

Based on these findings, this study aims to systematically assess hyperoside’s protective effects on liver pathology, lipid metabolism, and inflammation in high-fat, high-sugar diet-induced NAFLD rats. Using 16S rRNA sequencing and untargeted metabolomics, we further explore whether hyperoside’s therapeutic actions are associated with gut microbiota remodeling and regulation of serum metabolic profiles. This study aims to elucidate the mechanistic basis of hyperoside in NAFLD and provide critical experimental and theoretical evidence for its potential as a natural agent in NAFLD prevention and treatment.

Notably, hyperoside was specifically selected as a major bioactive flavonoid glycoside in Hugan Qingzhi Formula, possessing well-documented hepatoprotective, anti-inflammatory, and anti-fibrotic activities—yet its role in NAFLD, particularly via the gut–liver axis, has not been fully elucidated. Unlike single-omics studies that merely characterize microbiota or metabolite changes in isolation, integrating 16S rRNA sequencing with untargeted metabolomics allows for the identification of direct “microbiota–metabolite–liver” regulatory cascades, thereby offering novel mechanistic insights that are unattainable with single-omics strategies. This study addresses a critical gap in the field: while gut microbiota and metabolic dysregulation are well recognized to contribute to NAFLD, the specific role of hyperoside in remodeling these interconnected biological networks and the underlying molecular crosstalk between perturbed gut microbiota and dysregulated metabolites in NAFLD have not been systematically explored.

## 2 Materials and methods

### 2.1 Animals and grouping

All animal procedures were approved by the Animal Ethics Committee of of the Sixth People’s Hospital of Dongguan (Approval No. Z2023-020) and performed in accordance with the ARRIVE Guidelines (2020) and the National Guidelines for the Care and Use of Laboratory Animals (GB 14925–2010, China). Every effort was made to minimize animal suffering, and all euthanasia procedures were conducted under anesthesia to reduce pain, adhering to the 3R principle (Replacement, Reduction, Refinement).

Thirty-two SPF male Sprague–Dawley rats (6–8 weeks old, 180–220 g) were obtained from Suzhou SBF Experimental Animal Co., Ltd. They were housed in a specific pathogen-free (SPF) facility under controlled conditions: temperature (22 ± 2 °C), humidity (50 ± 5%), and a 12-h light/dark cycle, with free access to food and water. After 1 week of acclimatization, rats were randomly assigned to five groups: normal control (*n* = 7), NAFLD model (*n* = 7), NAFLD + low-dose hyperoside (Hyp-L, *n* = 6), NAFLD + high-dose hyperoside (Hyp-H, *n* = 6), and NAFLD + rosiglitazone (positive control, *n* = 6). The sample size for each group (*n* = 6–7) was chosen based on a previous NAFLD study (21) that adopted the same animal model and intervention design. This sample size is commonly used in related preclinical research and is sufficient to detect significant differences in primary outcome measures (e.g., liver index, serum ALT/AST levels, and hepatic steatosis scores) at a significance level (*α*) of 0.05.

### 2.2 NAFLD model induction and drug intervention

Rats with NAFLD were modeled by an 8-week high-fat, high-sugar diet, while controls received standard chow. At week 8, one rat from each of the control and model groups was euthanized by cervical dislocation, and liver tissues were harvested for hematoxylin and eosin (H&E) staining. Histological analysis revealed marked hepatic steatosis, confirming successful model induction.

Drug intervention was initiated at week 8 and continued for 4 weeks. Throughout this phase, the high-fat, high-sugar diet was maintained in all groups except the normal control. The treatment regimens were as follows:

Rosiglitazone group (NAFLD + Ros): Rosiglitazone was dissolved in physiological saline and administered by oral gavage at 5 mg/kg/day, at a volume of 0.1 mL per 100 g body weight, once daily.

Hyperoside low-dose group (NAFLD + Hyp-L): Hyperoside was dissolved in physiological saline containing 0.5% carboxymethylcellulose sodium (CMC-Na) and administered at 0.6 mg/kg/day, at 0.1 mL/100 g body weight, once daily.

Hyperoside high-dose group (NAFLD + Hyp-H): Hyperoside was prepared similarly and administered at 1.5 mg/kg/day, at 0.1 mL/100 g body weight, once daily.

Normal control and model groups received daily oral gavage of physiological saline and 0.5% CMC-Na solution, respectively.

### 2.3 Sample collection and processing

Body weight was measured at baseline (week 0), and subsequently at weeks 2, 4, 8 (end of modeling), 10 (after 2 weeks of intervention), and 12 (end of experiment) using an electronic balance (Mettler-Toledo, PL2002), with precision of 0.1 g. Following a 12-h fast, blood samples were collected at week 12 and processed to obtain serum, which was aliquoted and stored at −80 °C for the measurement of alanine aminotransferase (ALT), aspartate aminotransferase (AST), triglycerides (TG), total cholesterol (TC), and metabolomic analysis. Immediately after blood collection, rats were euthanized by cervical dislocation. Livers were promptly removed, washed with ice-cold saline to eliminate blood, dried with filter paper, and weighed to determine the liver index: Liver index (%) = (liver wet weight/body weight) × 100%.

Gross morphological features (color, texture, surface smoothness) were recorded. Liver tissues were divided into three parts: one was fixed in 4% paraformaldehyde (Guge Biotech, Wuhan, G1101) for 24 h, processed for paraffin embedding, and used for histological staining (H&E and Sirius Red). The other two parts were stored at −80 °C for RT-qPCR and other subsequent analyses.

Fresh fecal samples were obtained 12 h before euthanasia. Rats were placed individually in sterile metabolic cages, and feces (≥500 mg per rat) were gathered into sterile, nuclease-free tubes, rapidly frozen in liquid nitrogen, and stored at −80 °C for 16S rRNA gene sequencing to analyze gut microbiota.

### 2.4 Preparation of paraffin-embedded liver sections

Fixed liver tissues (4% PFA, 24 h) were washed with saline and dehydrated using a graded ethanol series (75% → 85% → 90% → 95% → 100% I → 100% II), with each step lasting 0.5–4 h. Tissues were then cleared in xylene via ethanol-xylene mixtures and pure xylene, followed by infiltration in paraffin at 60 °C for three cycles (1 h each). Tissue blocks were processed on a paraffin embedding station. Sections (4 μm) were sliced with a rotary microtome, floated on a water bath, adhered to coated slides, and baked at 60 °C for 3 h prior to staining.

### 2.5 H&E staining

Paraffin sections were deparaffinized in xylene and rehydrated through a descending ethanol series. Sections were stained with hematoxylin for 5 min, differentiated in hydrochloric alcohol, rinsed under running water for bluing, and counterstained with eosin for 2–8 s. After dehydration and clearing, sections were mounted with neutral balsam. Histopathological alterations were examined under a light microscope at 400 × magnification. Five non-overlapping fields per section were randomly selected for evaluation. Steatosis and inflammatory infiltration were graded following the 2020 NAFLD diagnosis and management guidelines (scale 0–3), where elevated scores reflect greater liver damage (22).

### 2.6 Sirius red staining

After deparaffinization and rehydration, sections were stained with hematoxylin for 5–10 min, then with Sirius Red solution for 15–30 min (in the dark). Following distilled water washes, tissues were dehydrated, cleared in xylene, and mounted. Collagen fibers appeared red under bright-field microscopy. Images were acquired with an inverted microscope, and collagen area fraction was measured using Image-Pro Plus 6.0 by analyzing five random, non-overlapping fields per section to objectively assess hepatic fibrosis.

### 2.7 Serum biochemical assays

Serum ALT, AST, TG, and TC levels were measured using an automatic biochemical analyzer (Hitachi 7,180) and commercial kits (Nanjing Jiancheng Bioengineering Institute) following the manufacturer’s protocol. Briefly, thawed serum was centrifuged at 4 °C, combined with specific reagents, and incubated at 37 °C for 5 min; absorbance was read at 510 nm (ALT, AST) or 490 nm (TG, TC). The analyzer automatically calculated the concentrations based on standard curves.

### 2.8 Quantitative real-time PCR (qRT-PCR)

Total RNA was isolated from 100 mg of liver tissue using a spin-column RNA extraction kit. Briefly, tissue was homogenized in lysis buffer, phase-separated with chloroform, and the aqueous phase was collected. RNA was purified via column-based binding and washing steps, then eluted in RNase-free water. Concentration and purity were determined using a micro-spectrophotometer by measuring A260/A280 ratios (acceptable range: 1.8–2.0).

Using a reverse transcription kit, 1 μg of total RNA was reverse-transcribed into cDNA in a 20 μL reaction, which was then stored at −20 °C.

Quantitative PCR was performed using SYBR Green chemistry with gene-specific primers and cDNA template. Primers were designed with Primer Premier 5.0 (Table 1). Amplification conditions were: 95 °C for 10 min; 40 cycles of 95 °C for 20 s and 55 °C for 20 s; followed by a melt curve analysis. All samples were assayed in triplicate. Gene expression was determined using the 2^-ΔΔCT^ method with Gapdh as the internal control.

**Table 1:** Primer sequences for RT-qPCR.

Gene	Forward primer (5′–3′)	Reverse primer (5′–3′)
Tnf-α (RAT)	CACCATGAGCACGGAAAGCA	GCAATGACTCCAAAGTAGACC
Tlr4 (RAT)	GTTGGATGGAAAAGCCTTGA	CCTGTGAGGTCGTTGAGGTT
α-SMA (RAT)	GAAGAGGAAGACAGCACA	CATCATCACCAGCAAAGC
Gapdh (RAT)	AAGCCCATCACCATCTTCCA	ATGGCATGGACTGTGGTCAT

### 2.9 16S rRNA gene sequencing and gut microbiota analysis

Fecal genomic DNA was isolated from 200 mg samples using a commercial DNA extraction kit (Tiangen Biotech, DP328). Briefly, samples were homogenized in Buffer SL via bead beating (60 Hz, 1 min), incubated at room temperature for 5 min, and centrifuged (12,000 rpm, 4 °C, 2 min). Supernatants were transferred to new tubes, mixed with Buffer AL, incubated at 70 °C for 10 min, and then combined with ethanol. DNA was purified on spin columns (the per manufacturer’s protocol), eluted, and stored at −20 °C.

The 16S rRNA V4–V5 region was amplified with primers (F: AYTGGGYDTAAAGNG; R: TACNVGGGTATCTAATCC).

PCR was carried out in a 25 μL volume with Taq PCR Master Mix, gene-specific primers, and DNA template per the kit’s protocol. Cycling parameters were: 95 °C for 5 min; 30 cycles of 95 °C for 30 s, 55 °C for 30 s, 72 °C for 30 s; and a final extension at 72 °C for 10 min. Amplified products were confirmed by 1.5% agarose gel electrophoresis, purified with a gel extraction kit (Omega, D2500-02), quantified, and pooled in equimolar ratios. Sequencing was performed by Majorbio on an Illumina MiSeq platform.

Raw sequencing reads were filtered to eliminate low-quality sequences (*Q* < 20), adapters, and chimeras. High-quality sequences were clustered into operational taxonomic units (OTUs) at 97% similarity using UPARSE (v7.0). Alpha diversity (Chao1 and Shannon indices) was computed with Mothur to evaluate microbial richness and diversity. Beta diversity was assessed via principal coordinates analysis (PCoA) in R (v4.0.3). Taxonomic assignment was conducted using the RDP Classifier against the Silva database. Bar plots and heatmaps were used to visualize relative abundances at the phylum and genus levels. Venn diagrams in R illustrated shared and unique OTUs across groups.

### 2.10 Untargeted metabolomics analysis

Serum samples (50 μL) were thawed at 4 °C and centrifuged (12,000 rpm, 4 °C, 10 min) to remove particulates. Using 200 μL of ice-cold methanol-acetonitrile (1:1), proteins were precipitated via 30 s vortexing, 30 min incubation at 4 °C, and centrifugation (12,000 rpm, 15 min, 4 °C). The supernatant was transferred to a fresh tube, dried under vacuum in a freeze dryer (BoYikang, FD-1A-50) for 4 h, and reconstituted in 100 μL of methanol–water (1:1, v/v). Following vortexing and centrifugation, the solution was filtered through a 0.22 μm organic membrane and analyzed promptly.

Metabolite profiling was performed using ultra-performance liquid chromatography coupled with tandem mass spectrometry (UPLC-MS/MS; Waters ACQUITY UPLC I-Class/Xevo TQ-S). Chromatographic separation was achieved on an ACQUITY UPLC BEH C18 column (2.1 mm × 100 mm, 1.7 μm) at 35 °*C. mobile* phase A was 0.1% formic acid in water, and mobile phase B was 0.1% formic acid in acetonitrile. The gradient conditions were: 0–2 min, 5% B; 2–10 min, 5–95% B; 10–13 min, 95% B; 13–13.1 min, 95–5% B; 13.1–16 min, 5% B; flow rate: 0.3 mL/min; injection volume: 2 μL.

Mass spectrometry was conducted in electrospray ionization (ESI) mode with concurrent positive and negative ion detection. Settings included: capillary voltage, 3.0 kV (+)/2.5 kV (−); source temperature, 120 °C; desolvation temperature, 350 °C; desolvation gas flow, 800 L/h.

Progenesis QI was used to process metabolomic data, including peak detection and normalization. Differential metabolites were identified by multivariate analysis (SIMCA 14.1; VIP > 1.0, *p* < 0.05) and annotated using HMDB/Metlin. KEGG pathway analysis was subsequently performed in MetaboAnalyst 5.0. Heatmaps of differential metabolites were generated using R.

### 2.11 Statistical analysis

Data are presented as mean ± standard deviation. Statistical analyses were carried out using SPSS 26.0 and GraphPad Prism 9.0. Multiple group comparisons for general indicators (e.g., body weight, liver index, serum biochemistry, gene expression levels) were performed using one-way ANOVA, with LSD-t tests for *post hoc* pairwise comparisons. A *p*-value < 0.05 was considered statistically significant. For OTU-level analysis (16S rRNA sequencing), multiple comparisons were controlled using the false discovery rate (FDR) correction (Benjamini–Hochberg method) with a threshold of *q* < 0.05 to reduce Type I errors. For metabolite-level analysis (untargeted metabolomics), differential metabolites were identified by combining VIP > 1.0 (from partial least squares discriminant analysis, PLS-DA) with FDR-corrected *p* < 0.05 (Benjamini–Hochberg method), ensuring robust selection of biologically relevant metabolites. Multivariate analysis was performed using PLS-DA in SIMCA 14.1. To avoid overfitting, a 7-fold cross-validation was applied to determine the optimal number of latent variables (LVs), and permutation tests (200 permutations) were conducted to validate the model’s reliability (permutation *p* < 0.05). No overfitting was observed as the cross-validated *R*^2^ and *Q*^2^ values were consistent with the original model, confirming the model’s robustness. Correlation analysis between gut microbial genera and serum metabolites was performed using Spearman’s rank correlation coefficient, with FDR correction applied to adjust for multiple comparisons (*q* < 0.05).

## 3 Results

### 3.1 Hyperoside alleviates body weight gain, liver index, and hepatic histopathological alterations in NAFLD rats

**Figure 1:**
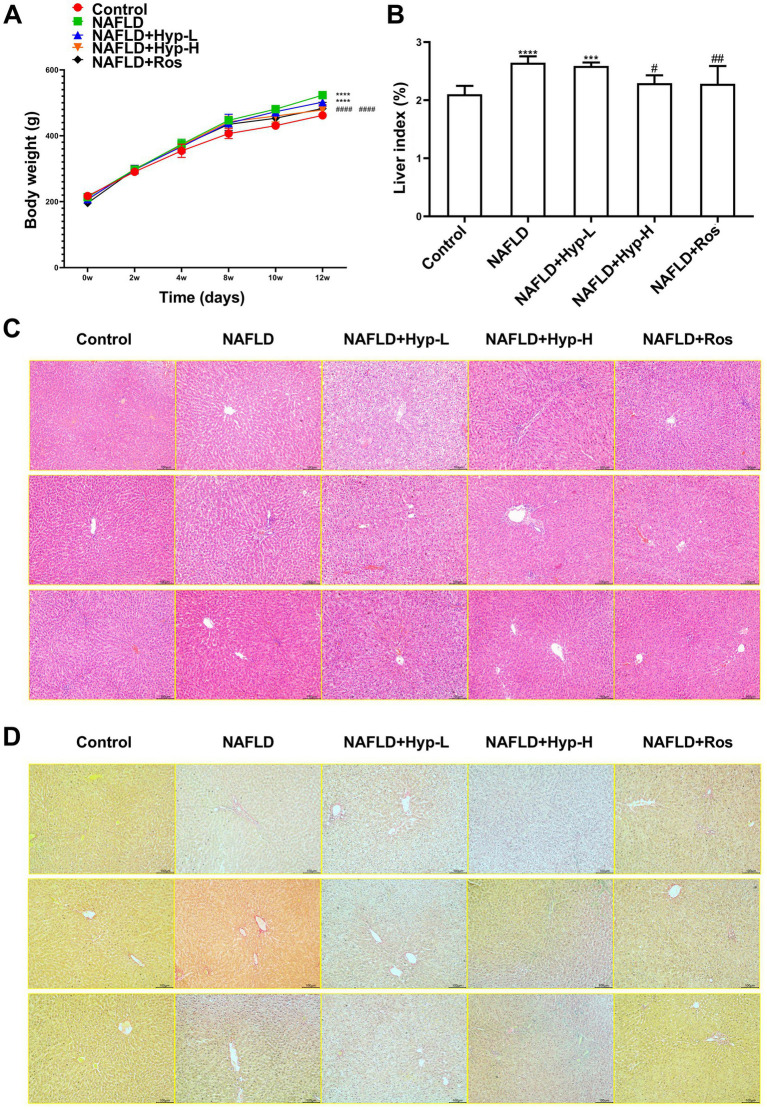
Effects of hyperoside on body weight, liver index, and histopathology of NAFLD in rats. **(A)** Trends in body weight changes. **(B)** Comparison of liver index. **(C)** Histopathological alterations shown by HE staining. **(D)** Degree of liver fibrosis shown by Sirius Red staining. The notation “*” indicates the difference compared to the control group (*p < 0.05, **p < 0.01, ***p < 0.001, ****p < 0.0001), while # denotes the difference compared to the model group (^#^*p* < 0.05, ^##^*p* < 0.01, ^###^*p* < 0.001, ^####^*p* < 0.0001).

To evaluate the therapeutic effects of Hyp on NAFLD, NAFLD was induced in rats via a 12-week high-fat, high-sugar diet, followed by intervention with two doses of hyperoside. Dynamic body weight monitoring showed that rats in the normal control group exhibited steady weight gain throughout the experiment (Figure 1A). From week 4 onward, the NAFLD model group gained significantly more weight, confirming successful obesity phenotype induction. Compared with the model group, both the low- and high-dose hyperoside groups exhibited markedly attenuated weight gain after week 8, with the high-dose group showing a more pronounced effect. The positive control group treated with rosiglitazone also demonstrated effective body weight control, suggesting that hyperoside exerts anti-obesity effects in a dose-dependent manner.

The liver index (liver weight/body weight ratio) is a key indicator of hepatic steatosis and inflammatory burden. The elevated liver index in the model group (Figure 1B) confirmed severe lipid accumulation. Hyperoside treatment significantly reduced the liver index in both low- and high-dose groups, with greater efficacy observed in the high-dose group. Histopathological examination corroborated these observations. H&E staining demonstrated that hepatocytes in the control group were regularly arranged with uniform cytoplasm and no lipid vacuoles (Figure 1C). In contrast, the model group exhibited extensive intracellular lipid droplets of varying sizes, hepatocyte swelling, and mild inflammatory infiltration-hallmarks of typical hepatic steatosis. Following hyperoside intervention, the low-dose group showed reduced numbers of lipid vacuoles and improved cellular architecture. In the high-dose group, hepatic steatosis was markedly ameliorated, with tissue morphology approaching that of the control group. The rosiglitazone group exhibited near-normal hepatic histology. Sirius Red staining was used to assess liver fibrosis (Figure 1D). Prominent fibrous septa formation in the model group, unlike minimal deposition in controls, confirmed early liver fibrosis. Hyperoside treatment reduced collagen deposition in the low-dose group, while the high-dose group showed almost complete suppression of fibrotic progression, with significantly decreased collagen area. This effect was comparable to that of rosiglitazone. Taken together, these results demonstrate that hyperoside effectively alleviates obesity-related phenotypes, reduces hepatic lipid burden, improves hepatocellular structure, and potently inhibits fibrosis progression, indicating multi-dimensional hepatoprotective effects.

### 3.2 Hyperoside improves liver function, alleviates lipid metabolic dysregulation, and modulates key inflammatory and fibrotic markers

**Figure 2:**
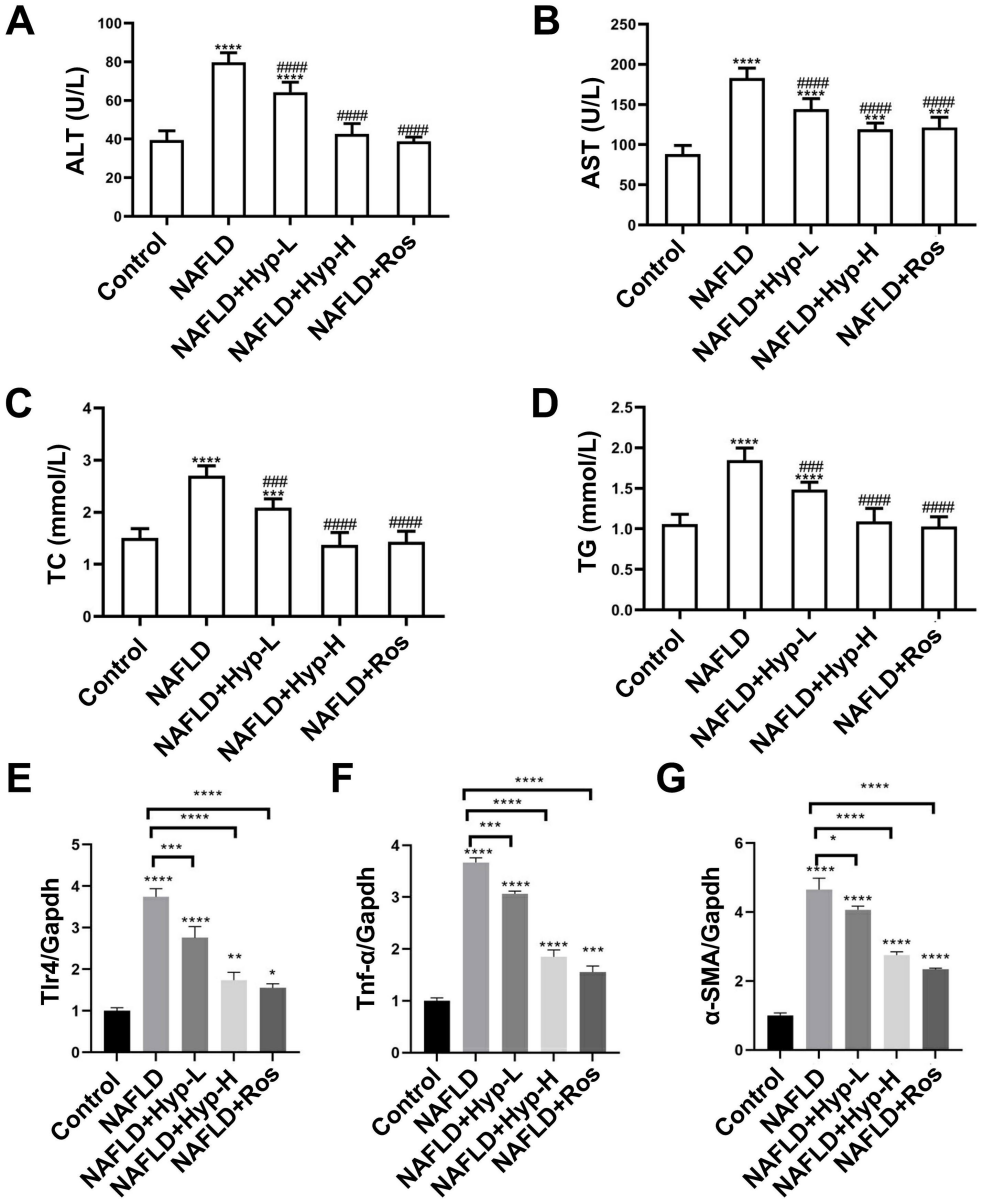
Effects of hyperoside on serum biochemical indicators and expression of liver fibrosis-related genes in NAFLD rats. **(A)** Dynamic changes in serum ALT levels. **(B)** Dynamic changes in serum AST levels. **(C)** Dynamic changes in serum TC levels. **(D)** Dynamic changes in serum TG levels. **(E)** Relative expression level of *Tlr4* mRNA in liver. **(F)** Relative expression level of α-SMA mRNA in liver. **(G)** Relative expression level of *Tnf*-α mRNA in liver. The notation “*” indicates the difference compared to the control group (*p < 0.05, **p < 0.01, ***p < 0.001, ****p < 0.0001), while “#” denotes the difference compared to the model group (^#^*p* < 0.05, ^##^*p* < 0.01, ^###^*p* < 0.001, ^####^*p* < 0.0001). Abbreviations: ALT, alanine aminotransferase; AST, aspartate aminotransferase; TC, total cholesterol; TG, triglyceride; NAFLD, non-alcoholic fatty liver disease; Hyp-L, low-dose hyperoside; Hyp-H, high-dose hyperoside; Ros, rosiglitazone; Tlr4, Toll-like receptor 4; *Tnf*-α, tumor necrosis factor-α; α-SMA, α-smooth muscle actin; Gapdh, glyceraldehyde-3-phosphate dehydrogenase.

To evaluate the effect of hyperoside on liver function, serum ALT (Figure 2A) and AST (Figure 2B) levels were measured. In the control group, both ALT and AST remained at low levels. The model group exhibited an increase in both enzymes, reflecting progressive hepatocellular injury. Hyperoside treatment, particularly at the high dose, significantly reduced ALT and AST levels, indicating a protective effect on hepatocytes.

In terms of lipid metabolism, the model group showed significant elevations in TC (Figure 2C) and TG (Figure 2D), indicative of severe metabolic dysregulation. The high-dose hyperoside group exhibited a significant lipid-lowering effect, with both TC and TG levels markedly reduced. The low-dose group showed a trend toward improvement, though less pronounced than the high-dose group. The rosiglitazone group also effectively reduced serum lipid levels.

To explore the anti-inflammatory role of hyperoside, qRT-PCR analysis revealed a significant upregulation of Toll-like receptor 4 (*Tlr4*) in the model group compared to controls (Figure 2E), implicating activation of innate immune signaling by gut-derived endotoxins. Hyperoside treatment significantly suppressed *Tlr4* expression in a dose-dependent manner. Additionally, the pro-inflammatory cytokine tumor necrosis factor-*α* (*Tnf-α*) was markedly upregulated in the model group. Both low- and high-dose hyperoside groups effectively downregulated *Tnf-α* expression, indicating potent anti-inflammatory activity (Figure 2F).

With regard to fibrosis, α-smooth muscle actin (*α-SMA*), a marker of activated hepatic stellate cells, was significantly elevated in the model group, indicating initiation of the fibrotic process. Hyperoside treatment, especially at the high dose, significantly inhibited *α-SMA* expression, with the low-dose group also showing moderate suppression. With an efficacy comparable to that of rosiglitazone (Figure 2G), hyperoside improved liver function and lipid metabolism through multi-targeted suppression of the *Tlr4/Tnf-α* axis and α-SMA-mediated fibrosis.

### 3.3 Hyperoside remodels gut microbial structure and diversity in NAFLD rats

**Figure 3:**
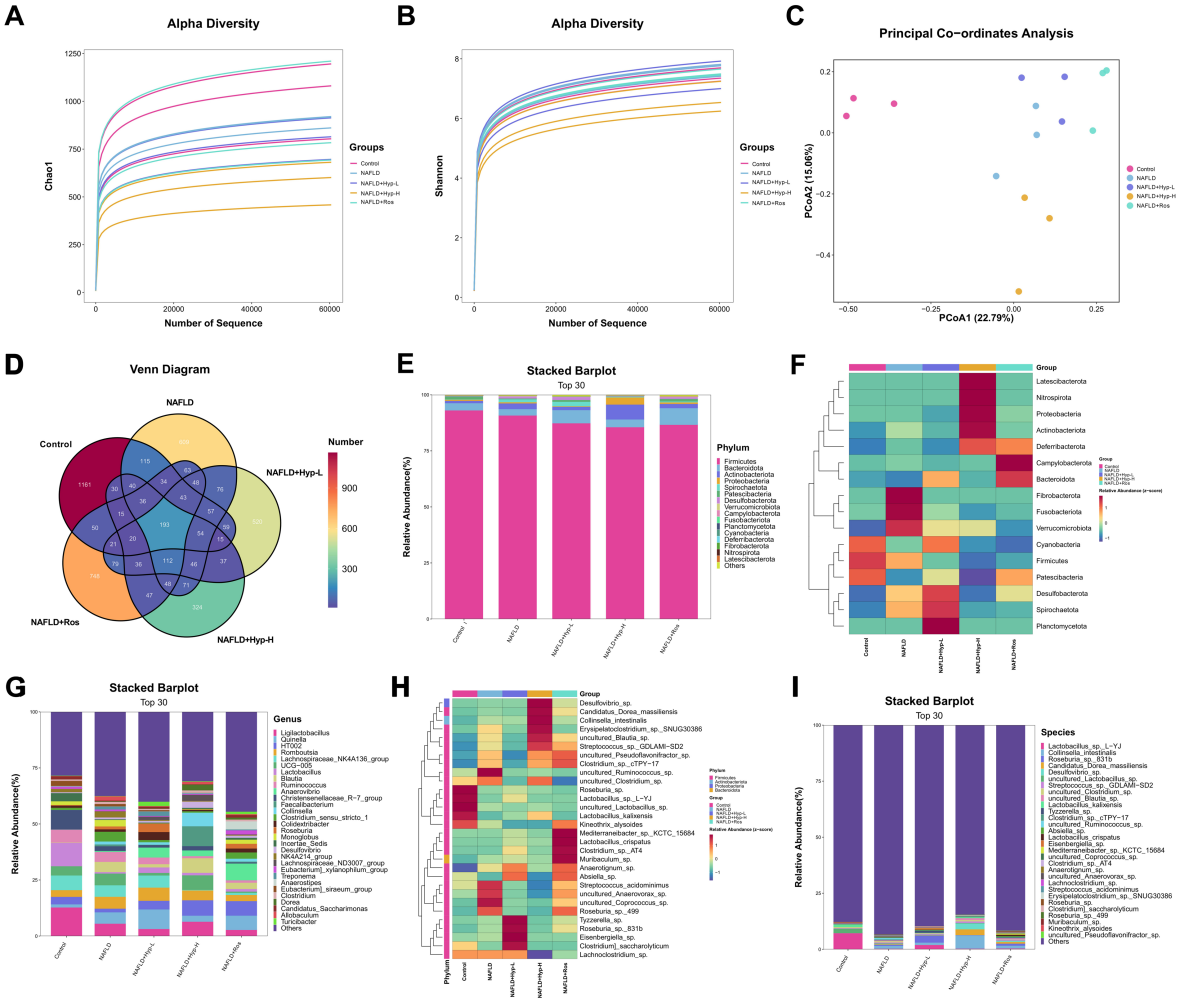
Impact of hyperoside intervention on the structure and diversity of gut microbiota in NAFLD rats. **(A)** α-diversity analysis reflected by Chao1 index. **(B)** α-diversity analysis reflected by Shannon index. **(C)** β-diversity differences revealed by PCoA. **(D)** Venn diagram analysis of shared and specific OTUs among different treatment groups. **(E,F)** Distribution and heatmap analysis of relative abundance at phylum level in gut microbiota (Top 30). **(G,H)** Distribution and heatmap analysis of relative abundance at genus level in gut microbiota (Top 30). **(I)** Distribution of relative abundance at species level in gut microbiota (Top 30).

Given the critical role of gut microbiota in NAFLD pathogenesis, fecal microbial composition was analyzed in all groups. Alpha diversity analysis revealed that the control and rosiglitazone groups exhibited the highest species richness (Chao1 index, Figure 3A) and relatively high community diversity (Shannon index, Figure 3B), indicating a stable microbial ecosystem. The model group showed the highest Shannon index but slightly lower Chao1 index, suggesting increased evenness but reduced overall complexity. Notably, the high-dose hyperoside group exhibited the lowest species richness and diversity, implying that high-dose intervention may selectively enrich specific taxa, leading to microbial simplification. The low-dose group displayed intermediate diversity, closer to the control group.

The PCoA revealed significant structural differences among groups (Figure 3C). The control and model groups were clearly separated, indicating that disease status profoundly altered microbial composition. The high-dose hyperoside group was most distinct from other groups, suggesting a strong remodeling effect on gut microbiota. The low-dose group partially overlapped with the model group, indicating a milder impact. The rosiglitazone group clustered closer to the model group.

Venn diagram analysis (Figure 3D) further illustrated the unique and shared features of microbial composition. The control group harbored the most unique OTUs (*n* = 1,161), suggesting a distinct ecological niche. The model group contained 609 unique OTUs, whereas the high-dose hyperoside group had only 324 unique OTUs but shared a relatively large number of OTUs with the control group, suggesting a shift toward a healthier microbial state. A core microbiome of 193 OTUs was shared across all five groups, potentially representing fundamental microbial taxa essential for host homeostasis.

At the phylum level (Figures 3E,F), Firmicutes were dominant in all groups, followed by Bacteroidota. However, the model group showed significant increases in Fusobacteriota and decreases in Fibrobacterota and Verrucomicrobiota, indicating potential pathobiont expansion and dysbiosis. The low-dose hyperoside group exhibited a recovery of Bacteroidota abundance and enrichment of beneficial metabolic taxa such as Desulfobacterota and Spirochaetota. The high-dose group showed increased Proteobacteria and reduced Firmicutes stability. The rosiglitazone group displayed a trend toward normalization, with decreased Firmicutes and increased Bacteroidota.

At the genus level (Figures 3G,H), *Ligilactobacillus* was the predominant genus across all groups. The control group was enriched with diverse lactobacilli (e.g., *Lactobacillus*_sp.-YJ, *uncultured_Lactobacillus*_sp.), maintaining a healthy microenvironment. In contrast, the model group showed reduced lactobacilli and increased abundance of potential pathobionts such as *Romboutsia* and *Clostridium_sensu_stricto_1*. The low-dose hyperoside group exhibited restored lactobacilli and enrichment of butyrate-producing genera such as *Tyzzerella*_sp. and *Roseburia*_sp. The high-dose group showed reduced lactobacilli but increased *Streptococcus_*sp._GDLAMI-SD2 and *Collinsella_intestinalis*. The rosiglitazone group showed partial recovery of certain lactobacilli (e.g., *L. crispatus*) (Figure 3I). Collectively, these results indicate that hyperoside significantly modulates gut microbial structure in NAFLD rats, with low-dose intervention promoting a lactobacilli-dominated healthy microbiota, while high-dose treatment may exert stronger therapeutic effects through selective enrichment of functional taxa.

### 3.4 Hyperoside regulates key functional genera and identifies potential biomarkers

**Figure 4:**
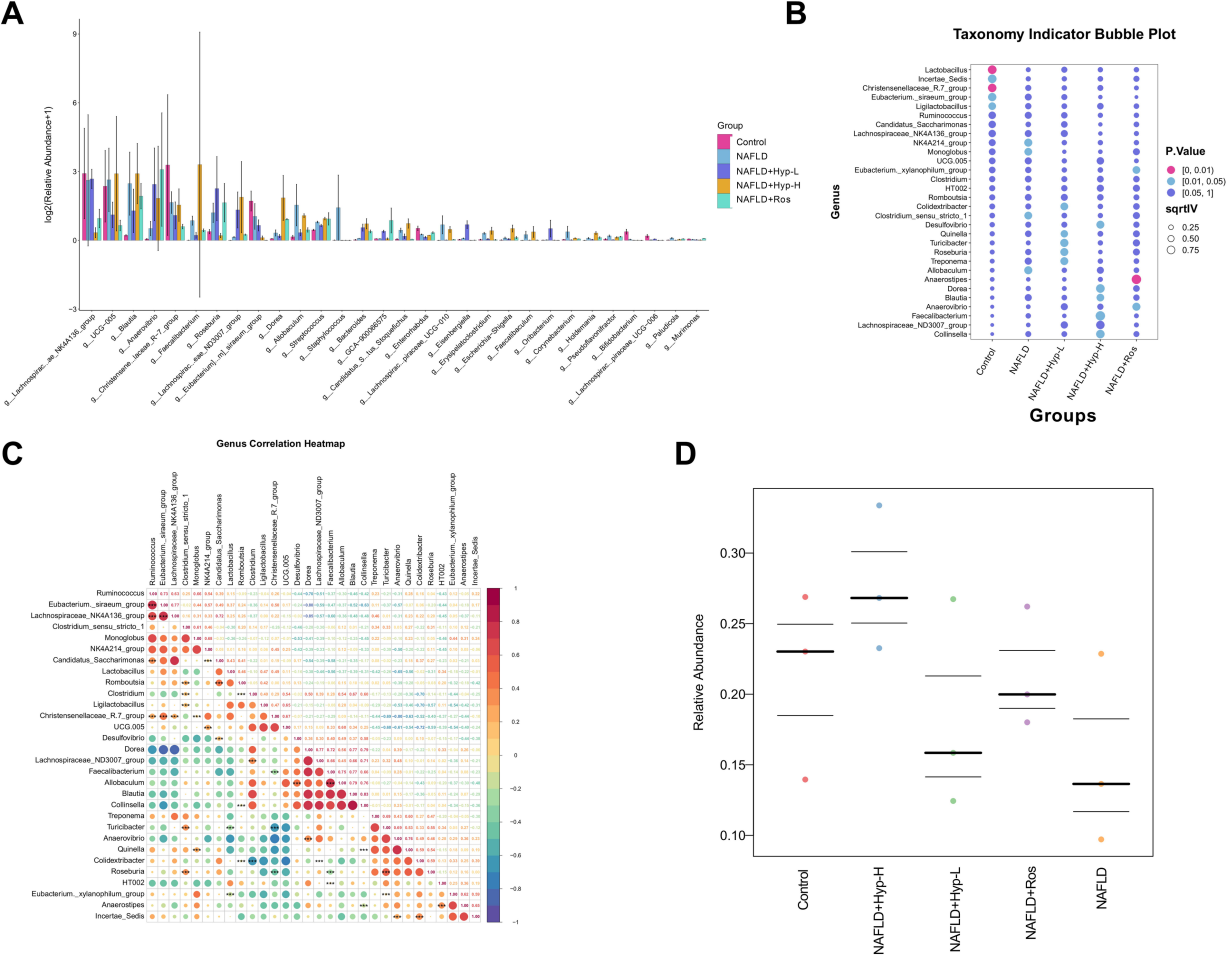
Differential analysis of key functional genera in gut microbiota and biomarker screening in NAFLD rats after Hyperoside intervention. **(A)** Heatmap of relative abundance at genus level in gut microbiota (Log_10_ transformed). **(B)** Taxonomy indicator bubble plot showing indicative species. **(C)** LDA score heatmap generated by LEfSe analysis (Top 50 genera). **(D)** Box plots of relative abundance of key genera.

To further dissect microbial functional changes, the top 30 differentially abundant genera were analyzed (Figure 4A). Butyrate-producing taxa such as *Lachnospiraceae_NK4A136_group*, *UCG-005*, and *Blautia* were relatively abundant across groups. Notably, these taxa showed a decreasing trend in the low-dose hyperoside group, possibly reflecting reduced host dependence on butyrate due to improved metabolism. In contrast, the model group exhibited slight increases in *Streptococcus* and *Escherichia-Shigella*, suggesting expansion of potential pathogens. At the genus level (Figure 4B), *Allobaculum* was significantly elevated in the model group but markedly reduced following drug intervention. Similar trends were observed for *NK4A214_group*, *Monoglobus*, and *Clostridium_sensu_stricto_1*. Correlation analysis (Figure 4C) revealed that genera sharing similar ecological functions, such as *fiber-degrading bacteria* (e.g., *Ruminococcus, Lachnospiraceae famil*y) or *SCFA-producing bacteria* (e.g., *Eubacterium*), often exhibited strong positive correlations, reflecting functional complementarity. In contrast, genera preferring distinct microenvironments (e.g., differences in pH, oxygen levels, or substrate types), such as *Dorea* vs. *Christensenellaceae*, or pairs of “beneficial vs. pathogenic bacteria” (e.g., *Faecalibacterium* vs. *Desulfovibrio*), typically displayed strong negative correlations, indicating ecological antagonism. Further analysis (Figure 4D) showed that the relative abundance of the target phenotype was significantly reduced in the model group, suggesting impaired gut microecological function under disease conditions. After intervention, especially in the high-dose group, the abundance of this phenotypic microbiota markedly increased. The Luo group also exhibited a favorable trend, suggesting the intervention positively enhances gut microecological function and restores core microbial composition. Hyperoside treatment, especially at the high dose, significantly reduced the abundance of these detrimental modules, restoring them to near-control levels. The rosiglitazone group also exhibited favorable regulatory trends. These findings suggest that hyperoside may restore overall gut microbial function by suppressing disease-associated taxa and rebalancing beneficial microbes.

### 3.5 Hyperoside significantly alters serum metabolomic profiles and regulates key metabolic pathways

**Figure 5:**
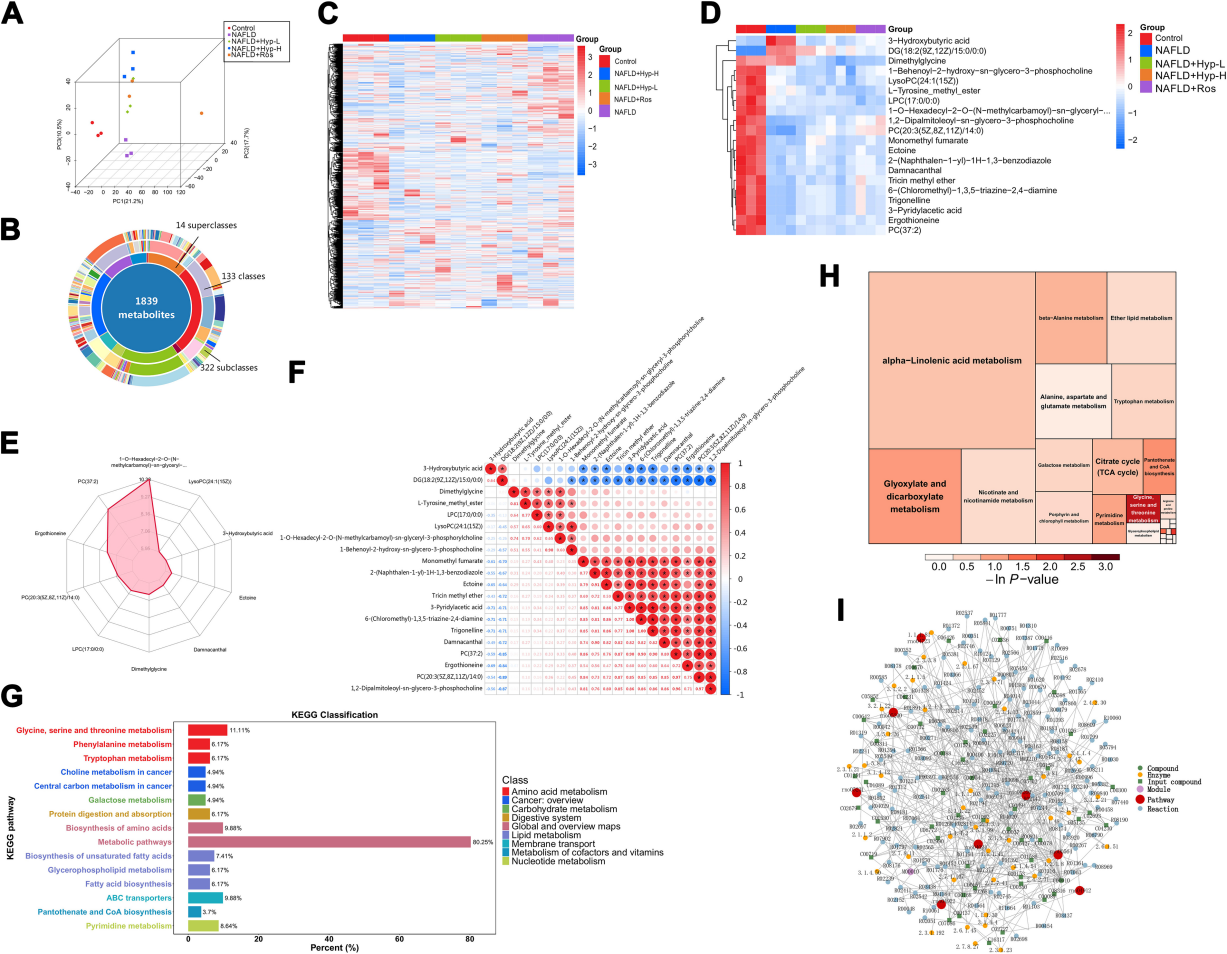
Effects of Hyperoside on serum metabolomics characteristics and metabolic pathways in NAFLD rats. **(A)** PCA principal component analysis. **(B)** Classification distribution of metabolites (donut chart). **(C)** Heatmap of metabolite expression profiles. **(D)** Heatmap displaying differential metabolite distribution. **(E)** PLS-DA discriminant analysis. **(F)** Volcano plot of differential metabolites. **(G)** KEGG pathway enrichment analysis. **(H)** KEGG function classification bar chart. **(I)** Metabolite-pathway network diagram.

To investigate host metabolic responses, untargeted metabolomics was performed on serum samples. PCA revealed that the control group samples formed a distinct and tight cluster, clearly separated from the samples of the other groups (Figure 5A). The model group deviated from the normal metabolic trajectory, while treatment groups—particularly the high-dose hyperoside group—trended toward re-clustering, indicating that hyperoside reverses metabolic disturbances. Three-dimensional PCA provided better resolution, with one outlier in the rosiglitazone group potentially reflecting individual variability. A total of 1,839 metabolites were identified, spanning 14 superclasses, 133 classes, and 322 subclasses (Figure 5B). Compared to the control group, the model group showed marked metabolic reprogramming, characterized by extensive up- and down-regulation across multiple pathways (Figure 5C). Heatmap analysis (Figure 5D) revealed significant enrichment of 3-hydroxybutyric acid (3-HB), DG(18:2/15:0), and dimethylglycine in the model group, which were notably reduced after hyperoside treatment. Radar plot analysis highlighted 1-O-Hexadecyl-2-O-(N-methylcarbamoyl)-sn-glyceryl and PC(37:2) as key discriminant metabolites (Figure 5E). Correlation analysis (Figure 5F) revealed that 3-HB and DG-class metabolites were negatively correlated with most other metabolites, suggesting their central roles in the metabolic network.

The KEGG pathway enrichment analysis (Figures 5G,H) indicated significant enrichment in “glycine, serine and threonine metabolism,” a pathway closely linked to one-carbon metabolism, antioxidant defense, and lipid synthesis. Additionally, the “alpha-linolenic acid metabolism” pathway contained the largest number of enriched metabolites, highlighting the importance of unsaturated fatty acid reprogramming in NAFLD. Regulatory network analysis (Figure 5I) revealed hub metabolites such as acetyl-CoA, citrate, and malate, which connect glycolysis, the TCA cycle, and fatty acid synthesis, forming a central metabolic nexus. Hyperoside-induced metabolic changes are associated with modulation of these key nodes, which may underlie its systemic regulatory effects.

### 3.6 The interaction between intestinal microbiota and metabolites

**Figure 6:**
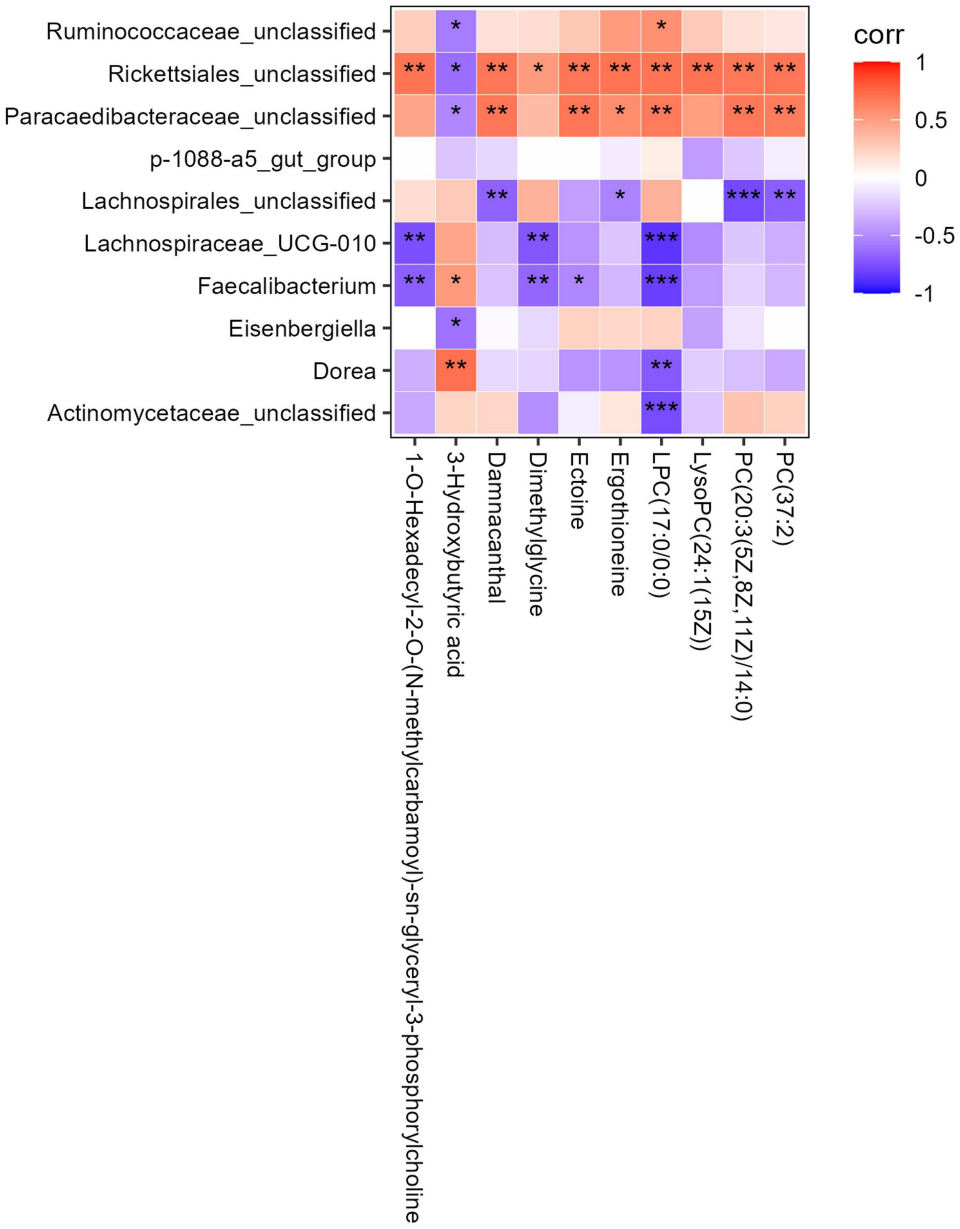
Correlation heatmap analysis between key genera in gut microbiota and serum metabolites.

To explore the “microbiota-metabolite-host” interactions, a correlation heatmap was constructed between genus-level gut microbiota and key differential metabolites (Figure 6). Beneficial genera such as *Faecalibacterium* and *Lachnospiraceae_UCG-010* were generally negatively correlated with pro-inflammatory metabolites [e.g., lysophosphatidylcholine (LPC), LysoPC] and ketone bodies (e.g., 3-HB), suggesting protective roles that are associated with reduced levels of harmful metabolites. In contrast, potential pathobionts such as *Rickettsiales_unclassified* and *Dorea* were positively correlated with lipid metabolites (e.g., DG and TG), indicating their association with increased lipid accumulation and inflammation, thereby exacerbating NAFLD progression. Notably, LPC and LysoPC emerged as common correlation targets for multiple bacterial genera, suggesting their role as key mediators linking gut dysbiosis to host lipid metabolic disturbances. Collectively, these results suggest that hyperoside may ameliorate systemic metabolic disorders by remodeling gut microbiota and regulating critical metabolic pathways—particularly amino acid and fatty acid metabolism—ultimately achieving multi-dimensional intervention in NAFLD.

## 4 Discussion

The pathogenesis of NAFLD involves intricate interactions between hepatic lipid accumulation, oxidative stress, chronic inflammation, and fibrogenic pathway activation, with increasing evidence underscoring the critical role of gut microbiota dysbiosis in promoting disease progression (23). Currently, no pharmacological agents are approved for NAFLD treatment, underscoring the urgent need for safe and effective interventions. This study systematically establishes that hyperoside, a natural flavonoid glycoside, confers multi-dimensional protection against HFD-induced NAFLD in rats. Its mechanism involves remodeling gut microbiota and reprogramming serum metabolites, thereby alleviating hepatic steatosis, inflammation, and fibrosis. Our findings provide compelling preclinical evidence for hyperoside as a promising microbiota-targeted therapeutic candidate for NAFLD.

Hyperoside ameliorated NAFLD phenotypes, reducing body weight, liver index, steatosis, and fibrosis. These phenotypic benefits were paralleled by significant reductions in serum ALT and AST levels, indicating effective protection against hepatocellular injury. Moreover, hyperoside effectively lowered serum TC and TG levels, demonstrating its potent capacity to correct systemic lipid metabolic disturbances. These findings align with prior studies emphasizing hyperoside’s antioxidant, anti-inflammatory, and glucose-lipid regulatory effects (24, 25). However, this study extends the pharmacological profile of hyperoside by demonstrating its comprehensive efficacy across the entire NAFLD spectrum—from steatosis to early fibrosis—thereby positioning it as a potential disease-modifying agent.

Mechanistically, we uncovered that hyperoside suppresses hepatic inflammation and fibrogenesis through modulation of key signaling pathways. Notably, NAFLD rats exhibited significant upregulation of *Tlr4* and *Tnf-α*, suggesting activation of the TLR4/NF-κB pathway, potentially triggered by gut-derived endotoxins such as LPS. Hyperoside intervention markedly inhibited this pro-inflammatory cascade, implying that it may interrupt the “gut-derived inflammatory signal” transmission to the liver. This effect could be mediated by enhanced intestinal barrier integrity or reduced colonization of pro-inflammatory bacterial taxa. Moreover, hyperoside markedly reduced α-SMA expression (26), a marker of activated hepatic stellate cells, especially at the high dose. This finding suggests that hyperoside not only halts but may even reverse early fibrotic processes, offering therapeutic potential beyond steatosis and into the more advanced NASH stage.

Our 16S rRNA sequencing results showed that hyperoside markedly alters gut microbiota composition in NAFLD rats. The model group exhibited dysbiosis characterized by elevated Shannon diversity but reduced Chao1 richness, along with expansion of potential pathobionts such as *Fibrobacterota* and *Fusobacteriota*. In contrast, hyperoside—particularly at the low dose—restored the dominance of beneficial *Lactobacillus* and enriched butyrate-producing genera such as *Roseburia* and *Tyzzerella*. These taxa are well-established for their roles in preserving gut barrier integrity, generating SCFAs, and regulating immune homeostasis (27–29). Venn analysis further revealed that the high-dose hyperoside group shared a relatively large number of core OTUs with the control group, suggesting a convergence of microbial community structure toward a healthier state. This microbial remodeling is likely associated with the restoration of host metabolic and immune equilibrium.

Notably, the high-dose hyperoside group manifested reduced alpha diversity (lower Chao1 and Shannon indices) yet the most distinct PCoA clustering and robust hepatoprotective activity, prompting pivotal inquiries into the ecological consequences of this microbial simplification. While reduced diversity is commonly intertwined with dysbiosis, the observed decline at the high dose likely reflects targeted enrichment of functionally pivotal taxa (e.g., metabolically beneficial bacteria) rather than deleterious dysregulation—corroborated by the simultaneous attenuation of hepatic pathological phenotypes and mitigation of pathobiont proliferation (e.g., *Romboutsia*, *Clostridium_sensu_stricto_1*). Regarding the resilience of this “simplified” microbial community, it has yet to be fully corroborated, but its shared core OTUs with the control group implies inherent stability in sustaining host metabolic homeostasis, though prolonged exposure could render it susceptible to ecological fragility. With respect to the trade-off between therapeutic efficacy and microbial diversity, low-dose hyperoside more closely mirrored the control microbiota in terms of diversity and composition, embodying a more favorable ecological configuration that harmonizes therapeutic potency with microbial ecological wholeness. Conversely, despite its therapeutic efficacy, high-dose hyperoside imposes more intense selective pressure on the gut microbiota, resulting in microbial simplification that necessitates prudence for prolonged use. Taken together, these findings illustrate that low-dose hyperoside strikes an optimal equilibrium between microbiota remodeling efficacy and ecological stability, whereas high-dose intervention prioritizes short-term hepatic improvements at the expense of reduced microbial diversity—underscoring the significance of dose refinement for clinical translation.

More importantly, untargeted metabolomics revealed that NAFLD induces a profound shift in the serum metabolome, characterized by aberrant accumulation of 3-HB, DGs, and dimethylglycine. 3-HB, a major ketone body, is typically elevated under conditions of enhanced fatty acid β-oxidation and energy imbalance. DGs are bioactive lipids strongly associated with insulin resistance and hepatic lipid deposition (30). Hyperoside intervention effectively normalized these dysregulated metabolites, indicating its ability to reverse systemic metabolic dysfunction. KEGG pathway enrichment revealed notable changes in “glycine, serine and threonine metabolism” and “alpha-linolenic acid metabolism.” The former is intricately linked to one-carbon metabolism, glutathione synthesis, and redox balance—key processes in oxidative stress defense (31, 32). The latter involves the synthesis of *ω*-3 polyunsaturated fatty acids, recognized for their anti-inflammatory and insulin-sensitizing properties (33). Thus, hyperoside may exert systemic metabolic reprogramming by targeting these critical metabolic nodes.

Crucially, correlation network analysis established a functional link between gut microbiota and serum metabolites, revealing a potential “microbiota-metabolite-liver” cascade. We found that beneficial genera such as *Faecalibacterium* and *Lachnospiraceae_UCG-010* were negatively correlated with pro-inflammatory lipids (e.g., LPC, LysoPC) and ketone bodies, while potential pathobionts (e.g., *Dorea*, *Rickettsiales_unclassified*) showed positive correlations with DGs and TGs. Notably, LPC and LysoPC are known activators of the TLR4/NF-κB pathway and inducers of hepatic inflammation (34, 35), which were identified as key hubs in this network. We therefore propose that hyperoside-induced enrichment of beneficial microbiota is associated with reduced levels of harmful metabolites like LPC, which may collectively contribute to ameliorated liver inflammation—though causal links require further validation, thereby forming a “gut microbiota → serum metabolites → hepatic inflammation” regulatory axis.

This study offers novel mechanistic insights into how hyperoside ameliorates NAFLD, demonstrating a multi-tiered pathway involving gut microbiota remodeling—characterized by enriched beneficial taxa (e.g., *Lactobacillus*, *Roseburia*) and suppressed pathobionts (e.g., *Romboutsia*, *Clostridium_sensu_stricto 1*)—, subsequent serum metabolic reprogramming, that normalizes dysregulated metabolites (e.g., 3-hydroxybutyric acid, diacylglycerols) and modulates key pathways (glycine/serine/threonine metabolism, alpha-linolenic acid metabolism), and consequent hepatic pathological improvement. Its effect goes beyond single-target inhibition; instead, it coordinately regulates lipid metabolism, inflammation, and fibrosis. Although this study did not establish causal relationships through functional validation (e.g., fecal microbiota transplantation or metabolite supplementation), the integrated 16S rRNA sequencing and untargeted metabolomics analyses reveal significant correlations between gut microbial alterations, serum metabolic shifts, and hepatic phenotype improvements—identifying key mediators (e.g., lysophosphatidylcholine) that link the gut–liver axis—thereby offering a rich set of candidate targets for future mechanistic investigations. A potential limitation of this study is the relatively small sample size, which may restrict the statistical power of subgroup analyses (e.g., correlations between gut microbiota and serum metabolites). Larger sample sizes in future studies could strengthen the reliability of these associative findings.

Future studies should prioritize: (1) identifying the microbial metabolites of hyperoside and their bioactivity; (2) validating the necessity of key bacterial taxa (e.g., *Lactobacillus*, *Roseburia*) in mediating hyperoside’s therapeutic effects; and (3) evaluating its long-term efficacy in advanced NASH or fibrosis models. Notably, hyperoside, as a naturally occurring flavonoid glycoside, exhibits favorable safety and bioavailability profiles, making it a strong candidate for development as a microbiota-modulating functional food or adjuvant therapy for NAFLD.

## 5 Conclusion

Hyperoside exerts dose-dependent protective effects against high-fat and high-sugar diet-induced NAFL D in rats by remodeling gut microbiota (enriching beneficial taxa and suppressing pathobionts) and regulating serum metabolic networks (targeting glycine/serine/threonine and alpha-linolenic acid metabolism), supporting its potential as a multi-target therapeutic candidate for NAFLD.

## Data Availability

The original contributions presented in the study are included in the article/supplementary material, further inquiries can be directed to the corresponding authors.
